# People-centred primary health care: a scoping review

**DOI:** 10.1186/s12875-023-02194-3

**Published:** 2023-11-09

**Authors:** Resham B. Khatri, Eskinder Wolka, Frehiwot Nigatu, Anteneh Zewdie, Daniel Erku, Aklilu Endalamaw, Yibeltal Assefa

**Affiliations:** 1 School of Public Health, Faculty of Medicine, the University of Queensland, Brisbane, QLD Australia; 2 Health Social Science and Development Research Institute, Kathmandu, Nepal; 3 International Institute for Primary Health Care-Ethiopia, Addis Ababa, Ethiopia; 4 Centre for Applied Health Economics, School of Medicine, Griffith University, Southport, QLD Australia; 5 Menzies Health Institute Queensland, Griffith University, Southport, QLD Australia; 6 College of Medicine and Health Sciences, Bahir Dar University, Bahir Dar, Ethiopia

**Keywords:** People-centred care, Integrated care, Primary health care, Primary care

## Abstract

**Background:**

Integrated people-centred health services (IPCHS) are vital for ensuring comprehensive care towards achieving universal health coverage (UHC). The World Health Organisation (WHO) envisions IPCHS in delivery and access to health services. This scoping review aimed to synthesize available evidence on people-centred primary health care (PHC) and primary care.

**Methods:**

We conducted a scoping review of published literature on people-centred PHC. We searched eight databases (PubMed, Scopus, Embase, CINAHL, Cochrane, PsycINFO, Web of Science, and Google Scholar) using search terms related to people-centred and integrated PHC/primary care services. We followed the Preferred Reporting Items for Systematic Reviews and Meta-Analyses Extension for Scoping Reviews (PRISMA-ScR) checklist to select studies. We analyzed data and generated themes using Gale's framework thematic analysis method. Themes were explained under five components of the WHO IPCHS framework.

**Results:**

A total of fifty-two studies were included in the review; most were from high-income countries (HICs), primarily focusing on patient-centred primary care. Themes under each component of the framework included: engaging and empowering people and communities (engagement of community, empowerment and empathy); strengthening governance and accountability (organizational leadership, and mutual accountability); reorienting the model of care (residential care, care for multimorbidity, participatory care); coordinating services within and across sectors (partnership with stakeholders and sectors, and coordination of care); creating an enabling environment and funding support (flexible management for change; and enabling environment).

**Conclusions:**

Several people-centred PHC and primary care approaches are implemented in HICs but have little priority in low-income countries. Potential strategies for people-centred PHC could be engaging end users in delivering integrated care, ensuring accountability, and implementing a residential model of care in coordination with communities. Flexible management options could create an enabling environment for strengthening health systems to deliver people-centred PHC services.

**Supplementary Information:**

The online version contains supplementary material available at 10.1186/s12875-023-02194-3.

## Introduction

The concept of “integrated and people-centred care” comprises two overarching concepts: integrated and people-centred care. The first concept, integrated care, is advanced from conventional illnesses-oriented and disease-focused health care. Illnesses-oriented care focuses on illness and cure, episodic consultation, and users as consumers purchase care. In contrast, disease-focused care refers to the management of diseases and priority disease control interventions, including their risk factors [1]. Additionally, integrated care means putting people and communities (not diseases), at the centre of health systems and empowering people and communities to take charge of their health by ensuring well-coordinated care around their needs, responding to fragmentations of care, and improving quality and cost-effectiveness rather than being passive recipients of services [1, 2].

Furthermore, integrated care emphasizes holistic care to improve population health and wellbeing with continued care across the life course, around needs with shared responsibility and accountability [3]. Ensuring integrated care empowers people to tackle the determinants of ill-health through systems thinking and partnerships, encouraging them to become co-producers of care in multilevel (individual, organizational and policy) systems [3]. Thus, integrated care is best understood as a set of practices intricately shaped by contextual factors to improve health status, and reduce morbidities and mortalities [4].

Moreover, the second concept, i.e., people-centred care (PCC) is derived from patient and person-centred care. In the late 1960s, patient-centred care (different from illness-oriented care) was introduced and continued for several decades, opposing previously prevailing bio-medically oriented and paternalistic views of healthcare [5]. Patient-centred care aims to make a functional life, affirming the ethical principles of respect for persons and justice, striving to make the health system more responsive to the health services needs [5, 6]. Advocates of market solutions to healthcare have been adopting patient-centred care by arguing for improved flexibility of consumer-oriented health care options and enhancing individual choice [7]. In contrast, person-centred care refers to caring for a meaningful life, and is a further development of patient-centred care based on personal philosophy, where the person denotes human and distinguishes from everything else [5]. Primarily, PCC is an expansion of patient-centred/person-centred care where people are involved in a care cycle, including the public, healthcare practitioners, and care organizations or systems. The PCC focuses on organizing principles for integrated care as a service innovation relating to individual service users, families and concerned communities [2]. Transforming the health care system towards people-centred health care requires action at four levels of the system: i) individuals, families and communities; ii) care providers; iii) health organizations; and iv) health systems [8]. The PCC is associated with better care continuity, considered care delivery by frontline workers within the health system, and responsive care practices and service utilization [9, 10].

The World Health Organization’s (WHO) Framework on integrated people-centred health services (IPCHS) combines the concepts of integrated care and people-centred care [11]. The framework envisions that all people have equal access to quality health services, co-produces health care to meet their health needs across the life course and respect their preferences, and coordinated and quality care (comprehensive, safe, effective, timely, efficient, and acceptable) along the continuum by all skilled and motivated carers and work in a supportive environment [11]. The conceptualization of integrated PCC puts people’s needs first in designing and delivering health services with principles of quality, safety, longitudinality (duration and depth of contact), closeness to communities, and responsive care (equity in access, quality, responsiveness and participation, efficiency, and resilience) [12]. Specifically, the WHO framework on IPCHS outlines five interwoven strategies for management and health service delivery: engaging and empowering people and communities; strengthening governance and accountability; reorienting the model of care; coordinating services within and across sectors; and creating an enabling environment and funding support [13, 14].

Primary health care (PHC) is a whole-of-society approach to organize and strengthen national health systems to bring health services closer to communities. The PHC approach comprises integrated health services to meet people’s health needs throughout their lives, addressing the broader determinants of health through multisectoral actions and empowering communities to improve health [15]. While primary care is a first level of care, it is usually delivered from prehospital, peripheral health facilities, and community settings [3]. People-centred PHC is the foundation of health systems that prioritize people first and have the potential to address diverse health needs by putting people and communities at the center of the system, empowering personalized health decision-making, and adapting health services to the local socio-cultural context [16]. Current body of literature focuses on people-centred integrated health services, especially medical care in hospitals, or family medicine or care by general practitioners. Nonetheless, there is a dearth of research that synthesize standalone studies on people-centred PHC and primary care using the WHO’s IPCHS framework. Thus, this study aimed to synthesize evidence on people-centred PHC interventions and strategies, their issues, and challenges. The findings of this review could inform strategies for strengthening the health system towards people-centredness in PHC systems and delivery and utilization of services.

## Methods

This study is a scoping review of the literature reporting people-centred PHC services/ primary care. A scoping review method helps to synthesize and analyze existing literature on a topic and map the scope of available evidence. The process involves six steps: identifying the research question; identifying relevant studies, selecting studies; charting data; collating, summarizing, and reporting results; and consultation (optional) [17, 18]. We employed the Preferred Reporting Items for Systematic Reviews and Meta-Analyses Extension for Scoping Reviews (PRISMA-ScR) checklist to support comprehensive reporting of methods and findings (Supplementary Information, Table S1) [19, 20].

### Identifying the research question

We identified the research question focusing on people-centred PHC/primary care services. The key research question was to review and synthesize the evidence on issues and challenges related to people-centredness in PHC/primary care services. We brainstormed on two concepts: people-centred care and PHC/primary care. These concepts guided identifying search terms under each concept and developing search strings. Our research team assumed that the proposed research question is broad to provide a breadth of issues to be explored in the review. The research question was further clarified by preliminary discussion among authors and agreed on the scope and significance of the topic.

### Identifying relevant studies

We searched eight databases (PubMed, Scopus, Embase, CINAHL, Cochrane, PsycINFO, Web of Science, and Google Scholar). The search strategy was built on two key concepts and related search terms: People-centred care (patient centred care, people centred care, person centred care, patient-centred care, people-centred care, person-centred care, patient centered care, people centered care, person centered care, patient-centered care, people-centered care, client-centered care, client centered care, person-centered care); Primary Health Care (primary health care, public health care, community care, primary care, primary care nursing, family medicine, family practice, general practice) on each database. Boolean operators (AND/OR) and truncations (“”, *) varied depending on the database. The search included all studies published in English until 30 January 2023 (no starting date was applied in the search). No country-related limitations were applied.

### Selection of studies

We included all studies that dealt with PCC regardless of their designs. Based on the title and abstract, screening was undertaken initially by the first author and further assessed by the second author. This was followed by a full-text screening initially by the first author and evaluated by the second and third authors. Any disagreements were resolved by discussion with the last author. We applied some post hoc inclusion and exclusion criteria based on the research question and new topic familiarity through reading the studies. For example, we included studies considering the population (health service users, care providers and managers), concept (PCC/integrated care), and contexts (PHC and primary care systems) of the study [21]. We included studies if their findings can answer our review question rather than the quality of individual studies. We followed the standard scoping review PRISMA-ScR checklist [19, 22] and took reference to previous scoping reviews [23, 24]. The included studies are based on the findings and their interpretation rather than the inclusion criteria [25, 26].

### Charting the data

A data-charting form was developed to extract data from each study covering author, year, country, type of study, key concepts, and main findings (Supplementary file, Table S2). Data were extracted by the first and double-checked by the second and last authors.

### Collating, summarizing, and reporting results

The first author did data analysis with guidance and support from the last author. Thematic analysis of data was conducted by adopting Gale’s framework method [27]. This analysis method adopts multiple steps such as collection of raw data (main findings about the research question for this review), familiarisation with data, paraphrasing of data/label according to the nature of data, developing/applying the analytical framework, charting data into the framework matrix, and finally interpretation. After reading and familiarisation the data, we extracted important concepts/categories and grouped them (with similar ideas) into the five components (engaging and empowering people and communities; strengthening governance and accountability; reorienting the model of care; coordinating services within and across sectors; creating an enabling environment and funding support) of the WHO ICPHS framework. Within each component, themes were generated by grouping similar categories/ideas and concepts. Findings were reported in three forms; first, outcomes of database search results were presented in the flow chart. Second, a customized summary of the data charting table (covering the author, location, and key ideas related to the research question) was presented. Finally, generated themes were explained and interpreted in the narrative paragraphs under each component of the analytical framework.

## Results

**Fig. 1:**
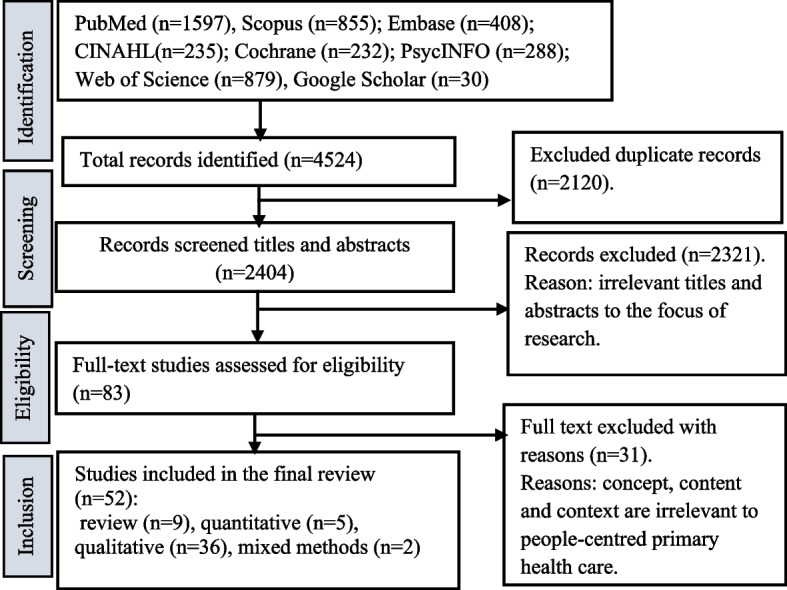
PRISMA-ScR flow chart showing the selection of studies for the review

A search yielded 4494 records from all databases (Fig. 1). We removed 2090 duplicated records. Then, studies were screened for relevance based on title and abstract, whereby 2321 were excluded, leaving 83 studies for full-text screening. A further 31 studies were excluded after the full-text screening with reasons. A further 52 studies were included in the final review.

### Overview of included studies and generated themes

Table 1 presents an overview of studies included in the review, including countries where studies were undertaken, and each study mapped with generated themes. Of 52 studies, 39 were from high-income countries (HICs): 19 studies were from the USA, eight studies were from Canada (4) and the Netherlands (4), six studies were from the UK (3) and Australia (3), four studies were from Norway (2) and Sweden (2), and one each from Greece and Finland. Two studies were from upper-middle-income countries (UMICs), including one from multi-country (Latin America), Mexico, and South Africa. Three studies were from low-and lower-middle-income countries (LMICs), including one from Nigeria and two from Uganda, and seven studies were not specified. Of five strategic components of the ICPHS framework (Table 1), 26 studies described engaging and empowering people and communities (light black); 32 studies included strengthening governance and accountability (blue); 45 studies explained reorienting the model of care (pink); 34 studies incorporated coordinating services within and across sectors (yellow); and 33 studies discussed creating an enabling environment and support for funding (green).

**Table 1:**
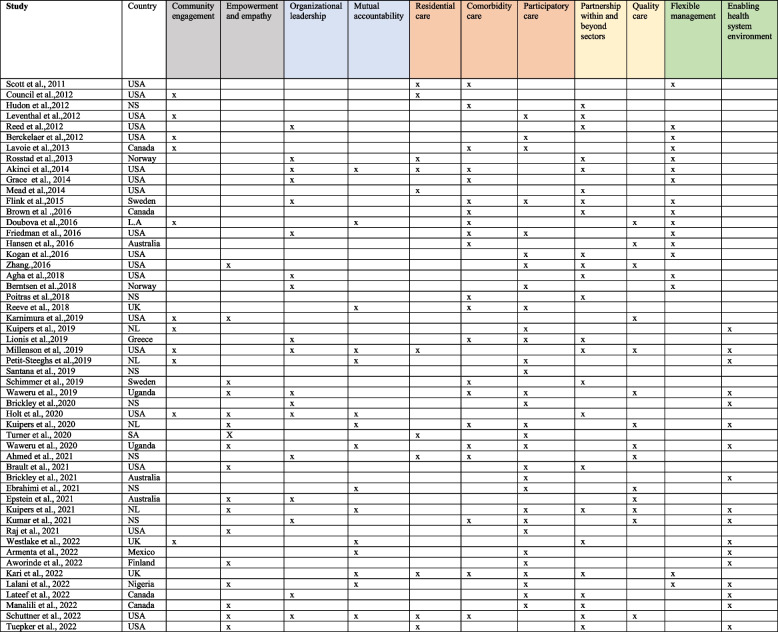
Summary of themes under the WHO framework on integrated people-centred health services [28–79]

*NS* Not specified, *NL* Netherland, *SA* South Arica, *LA* Latin America

Light black: themes under “engaging and empowering people and communities”

Blue: themes under “strengthening governance and accountability”

Pink: themes under “reorienting the model of care”

Yellow: themes under “coordinating services within and across sectors”

Green: themes under “creating an enabling environment and funding support”

### Engaging and empowering people and communities

Empowering and engaging people provides the opportunity, skills and resources needed to be articulated and empowered end users of health services and advocates for a reformed health system [13]. Two themes are described in this component: community engagement in health care (11 studies) and empathic empowerment (17 studies) related to PHC/primary care services.

#### Community engagement

The use of the PCC practices facilitated an ongoing relationship between end users (providers and service users) through a team relationship, timely communication, and care plan (e.g., enhanced coordinated, comprehensive care) [29, 33]. Additionally, peers promoted norms to other service users and worked together to improve routine care practices [31, 34]. Reinforcement of people engagement and positive perception improved people-centred primary and hospital care [41, 54, 59]. Contextual factors influencing PCC included perceptions of involvement, engagement, and co-creation of processes to achieve physical and social well-being for persons with multimorbidity [50, 51, 71]. Nonetheless, only the positive perception of the public filled the limited expectations, and service users’ voices were less incorporated, which decreased the traditional system's authority [41, 53].

#### Empowerment and empathy

Empathy, communication in multicultural languages, people’s involvement in making decisions and designing and implementing solutions effectively empower individualized care for their health [57, 59, 61]. Empathic support, communication with their doctors, understanding of problems, providers’ skills, and management plans were required for improved satisfaction and the effect of interpersonal care [61, 62, 70]. Communication between service users and providers enhanced high perceived empathy in consultation, trust in relationships, and positive experiences and satisfaction [50, 62]. The trusted relationships with providers, their involvement in care treatment decision-making, and emotional support from family and friends found effective people-centredness in care delivery [45, 67, 68, 77]. Local arrangements for service integration, multi-professional teams, and co-location also supported building relationships for community empowerment [75, 79].

Nonetheless, weak communication between individuals and practitioners was reflected in daily care practice activities and had poor priority in service delivery [73, 75]. Factors of poor empowerment in care continuity were lack of flexible design, high administrative tasks, limited appointment time, poor autonomy, and unavailability of providers [56, 64]. Several factors, such as intra- and interpersonal (e.g., perceived reluctance to engage in care), and organizational (e.g., limited encounter time, lack of discussion, psychological issues with health workers), also influenced understating the problems and health service needs [60, 62, 78].

### Strengthening governance and accountability

Strengthening governance requires a participatory approach to policy formulation, decision-making and performance evaluation at multilevel health systems, from policy-making to the service delivery level [13].Two themes under this component were: organizational leadership (18 studies) and mutual accountability (15 studies).

#### Organizational leadership

Strategies to strengthen organisational leadership, human capital, and facilitating adaptive culture and innovation contributed to innovative PCC primary care services [32]. For instance, the people-centred medical home (PCMH) model created an enabling environment for delivering quality care, reduced care costs, and organizational needs, and incorporated people’s voices into governance and accountability for operations [36, 53, 59, 63]. The role of professional councils (e.g., nursing, or general practitioners’ organizations) could be instituted to measure people-centredness for the implementation of PHC [58, 76]. Similarly, increased local leadership, team communication, and high physician engagement with service users facilitated the successful implementation of people-centredness in PHC [37, 46]. Multiple stakeholders offered an opportunity for reform and gaining an inclusive vision of PCC in Uganda [57] and Greece [52]. Furthermore, the use of digital technology supported the functionality of clinical information aligning with organizational support, availability of community resources, clinician interactions, and gap payment funding models to incentivize care workers [42, 63, 67].

However, the people-centred integrated care process failed to identify long-term goals, provide shared long-term care, and monitor and evaluate health care delivery for people with multimorbidity [47]. Furthermore, organizational and policy impediments (e.g., state decisional capacity laws and financial crisis), lack of documentation or low priority also impacted the delivery of integrated PCC [35, 39, 52, 78].

#### Mutual accountability

Understanding the health system and integrating different dimensions of care ensured the changing needs of people with complex chronic illnesses [49, 54]. Integrated responsiveness and relative priority for the cultural change improved client and professional interaction towards organized care [54, 59, 63, 69]. The government policy in health system organizations assessed incentives for care coordination to meet complex needs [36, 41, 78]. Innovations and people-centeredness shaped the access to health facilities, costs, users’ perceived quality of care and expectations, and availability of free services [53, 62]. Furthermore, approaching interpersonal and coordinated multidisciplinary teamwork, consultation on preventive and promotive measures supported people receiving treatment, medical information, and skill mix care practice towards people-centred holistic care [60, 66, 71, 74].

Nonetheless, fragmentation, segmentation, limited funding, insufficient coverage, poor quality, ageing and chronic conditions, and lack of effectiveness and sustainability were multilevel challenges to achieving mutual accountability towards PCC [68, 72]. Other influencing factors of mutual accountability were limited understanding of professional identities, roles, and responsibilities in continuity of care and service integration [75].

### Reorienting the model of care

Reorienting the model of care means ensuring that efficient and effective health care services are designed, purchased and provided through innovative models of care that prioritize primary and community care services and the co-production of health [13]. Three themes generated in this component were: residential and home-based care (11 studies), care for people living with multiple chronic conditions or multimorbid conditions (21 studies), and participatory care (30 studies).

#### Residential and home-based care

The residential model of care, known as the patient-centred Medical Home (PCMH), is a new form of transformation in healthcare that offers an interprofessional model by connecting services and management in a primary care setting [28, 36, 61, 79]. The centrepiece of transformation for primary care in a residential health care model restored confidence in quality of care and resulted in reduced care costs of hospital-related outcomes [36, 63]. Such a model that was developed in iterative phases (e.g., planning, acting, observing, supporting and transforming care practices) met the needs of people’s priorities, improved holistic and more people-centred care in primary care, and addressed the health needs of disadvantaged communities [29, 38, 53, 74]. The residency-affiliated community group family medicine provided goal-directed care for people with complex health problems (functioning, social situation, support and empowerment, and care satisfaction). It ensured self-management at home (e.g., engaging with nurses, telehealth, medication plans, and interactions) [29, 35, 74, 78]. Positive effects of functional residential care improved satisfaction, informal and formal caregivers in clinical screening as high-risk groups, and delayed placement [63, 79]. However, home-based residential care was unsuitable for managing several disease-based care pathways and specialist care to address individuals' needs for people with substantial comorbidity [35].

#### Care for people with comorbidities

PCC brought the management of chronic diseases to a new dimension of care (legitimizing the illness experience, acknowledging service users’ expertise, offering hope and providing advocacy) [30]. People with multimorbidity viewed PCC as a well-coordinated, respectful, supportive care long-term management of medical problems and prevention and promotion through behaviour change interventions [36, 60, 62]. Approaches of PCC in the management of comorbidities were effective communication, information, knowledge sharing, understanding demonstration of provider’s multidimensional skills, and agreement about treatment plans [39, 57, 69]. Additionally, care from non-physicians found important in comorbidities; for instance, pharmacists provided direct care services, ensured access to community resources, assisted care transitions, and provided interprofessional education [28]. Diabetes specialist nurses expressed needs that diverge and converge for people with diabetes [56]. Developing training for health care providers for self-management interventions and self-care practices positively impacted people with chronic diseases [42, 48]. Such care practices brought the benefit of clinical care, active involvement in care, and shifting from disease-focused to people-centred PHC [34, 35, 49, 74]. Furthermore, the Family Health Team and multidisciplinary providers pursued continuity and care coordination, allowing site-specific program implementation and commitment to timely delivery of health services [37, 40]. Interdisciplinary teams and informal caregivers enabled people-centred medication therapy for older people, management services with continuous quality improvement initiatives, and inpatient family medicine service [28, 63, 78]. Nevertheless, understanding variations between GP practices and poor documentation of records of people-centeredness had challenges in applying evidence-based medicine [39, 43]. Additionally, some of the populations (e.g., migrants and refugees) were neglected in the management of chronic disease, had gaps in irregular care and providers, lack of information (medical history to solve health problems), and limited time spent with the people [41, 52, 78].

#### Participatory model of care

Designing participatory and holistic or whole-person care (e.g., respect and value, choice, dignity, self-determination, purposeful living), and had the potential to address multiple dimensions of care for wellbeing (e.g., physical, mental, and social needs) by knowing and confirming tailored health plan, inter-professional teamwork, and care provision in collaboration with families [31, 44, 51, 52, 64, 66, 74, 77]. Understanding the participatory approach of PCC informed quality of care (e.g., availability of medication, shorter waiting times, flexible facility opening hours, courteous health workers) for care for ageing problems [62, 65, 70]. The care process for people with social and health complexity (for health needs of older adults, and referral practice) was found effective in primary care to adapt peoples’ preferences [54, 70]. There was effective communication by using electronic health records to people with complex health issues that supported the involvement of people and families in health care (e.g., practice set-up, planning, and change in consultation) [39, 45, 49, 57, 69, 72]. Participation of people built trust through shifting the role of self-care based on medical knowledge and pragmatic experience of engagement in care process, and ensuring provider relationship and guidance [33, 34]. Participation of service users (e.g., obtaining feedback, engaging stakeholders, adapting PCC quality improvement for better quality care) improved service integration and practices [42, 75, 77].

Participatory and coordinated care enhanced joint working, fostering communication and professional cultures (shared beliefs and values) by exploring and prioritizing the problems (e.g., knowing the person, identifying problems, prioritizing care, treatment, evaluating decisions and implementation) [72, 73, 75]. Approaches to co-design and co-creation built trust, partnering with professionals and users, communities, and individuals experience [51, 66, 74, 77]. Strategies of participatory care included evidence-based decision practice, enhancing interdisciplinary team approach to continuity of care, developing training for providers, involvement of people in sharing experience (e.g., empathy in consultation, physical and social wellbeing), and providers' attitudes (open communication, caring behaviours) [48, 50, 51, 54, 58]. Furthermore, system responsiveness for quality care (e.g., affordable, coordinated, accessible) moved towards the long-term goal of universal access [38, 47, 72]. However, challenges such as the unavailability of family physicians, limited information and communication technology, and heterogeneity of people-centred quality improvement influenced the integrated people-centred primary care among disadvantaged populations (e.g., refugees) [52, 55]. In some public facilities, the care process was unseen and disrespected, lacking continuity, transition, and coordinated care [61, 68].

### Coordinating services within and across sectors

Coordination requires integrating care providers within and across health care settings, developing referral systems and networks among levels of care, and creating linkages between health and other sectors [13]. Two themes were described under this component: partnership with stakeholders and sectors (24 studies) and coordination for quality care (14 studies).

#### Partnership with stakeholders and sectors

Partnership with other sectors supports engagement in people-centred PHC. Involvement of stakeholders and sectors (e.g., trust, understanding of purpose, clarity of expectations, and power-sharing) facilitated priorities for care evaluation and treatment outcomes [71, 74, 76]. Developing partnerships and team-based approaches (appointment tool guide communication) with people experiencing complex diseases to reduce stigma, social and relational integration for care coordination, and self-management [35, 36, 64, 77, 79].

Communication technology support partnership with other sectors. Communication technology and resources support non-physician healthcare providers [38, 78]. Integrated health information technology was perceived as effective in the organization and management of chronic diseases, including the medical and care needs (discharge-related information sent from the hospital and care providers linking the care process) [32, 39, 40, 48]. Electronic resources supplemented clinic visits through direct communication with people and providers [64]. Information technology supported the development of ongoing partnerships in innovation and integrating medical and social care to manage chronic illnesses, research, and practice [30–32, 44, 52, 78].The development integration of technology (e.g., mhealth tools and high-tech and high-touch technology) supported in identifying and engaging high-risk populations [53, 64, 77].

More attention toward changing the organization of the electronic health records system streamlined documentation work of care visits/encounters [45, 56]. Improved application, user-focused optimization efforts and tool functionality enabled to address the issues of access, health service and health literacy [46, 59]. At the same time, clinicians adopted information technology with the perceived value of data sources enhanced the development of interventions for people living with multimorbidity [31, 48, 59]. Updated electronic health records data analytics incorporated organization-wide procedures (staff, time management, cultivating staff collaborations) and follow-up services in PHC settings [39, 46, 56]. However, coordination and partnership with stakeholders had challenges in healthcare organizations, including work practice discrepancies and lack of enforcement agencies [36, 56, 76]. Additionally, the potential of information challenges influencing PCC was the lack of data protection laws (including documentation and dissemination, time pressure, and conflicting financial incentives) that impeded the use of digital records in care [68].

#### Coordination and communication

Prerequisites in co-creating optimal health care practice with and for older people and their expectations influenced the implementation of biomedical and public health interventions and quality of care [62, 66]. Coordinated care supports user-driven healthcare decision-making for quality improvement (reducing cost, relationship with providers), a perceived measure of quality care [64, 67], common perception, and sustainable primary care models to ensure quality care for physical and emotional health [50, 67]. Engaged physician-service users communication found that professionals care (dignity, respect, prioritize, and individualized care) for people with multiple health needs [60, 78]. Furthermore, the coordinated care of frontline staff in communicating with other stakeholders can address social and economic issues to implement quality integrated care [53, 63], instead of describing the holistic/whole person and PCC approach. GPs’ narrow disease-specific focus of guidelines was inappropriate for addressing people’s needs and health priorities[43]. Challenges in designing and implementing PCC interventions that hindered the delivery of integrated care were lack of clarity around responsiveness and readiness, lack of information and coordination of care, lack of integrating electronic health records in work practice (preferences, information, and education) [41, 45, 57, 68, 69].

### Creating an enabling environment

To implement strategies of four categories, it is necessary to create an enabling environment that brings together all stakeholders to undertake transformational change [13]. Two themes under this component were: flexible management options (17 studies) and enabling environment (17 studies).

#### Flexible management for care

The flexibility of management can create an enabling environment for PCC. Practice stakeholders address the local needs expectations by redesigning health and social, professional cultures and flexible program implementation [37, 75]. Care transitioned from hospital to home toward high-quality care that reduced unnecessary walk-in clinics and emergency department coordinating relationship building (with end users or organizations) and enhanced pharmacy services [28, 36, 40, 42]. Organizational perspectives (cost-effectiveness and health care delivery processes) improved long-term goal-driven people-centred integrated care and increased people and providers relationships (including knowledge, and satisfaction) [32–34, 47]. The PCMH model operationalized health services by providing a feasible reform option and solutions to people's engagement [28, 36, 44, 46]. However, flexible management and implementation were influenced by challenges (lack of resources and training, excessive caseloads, poor data management responsibilities, lack of medical neighbourhood) and inconsistent implementation of practices [36, 37, 42, 46, 75]. Also, difficult communication and being invisible in the context of event-based quality of care frameworks were identified as gaps in primary care clinics [41, 47].

#### Enabling the health system environment

Health workforce attributes (including the responsibility of professionals) enabled sensitizing systems (continuous supervision, professional training, empowerment for leadership) focused quality of care improvement initiatives to bring improved clinical practice [69, 71, 72]. Collaborative works (between a personal network of family and practitioners), upgrading of providers for quality improvement resources, alignment measurement efforts, engaging champions, and need assessment (needs/priorities for people-centred measurement) facilitated identification and management of symptoms [72, 73, 76, 77]. Similarly, co-location of community health systems, organizations, and service delivery outlets found committed care boundaries that provided sufficient care responsive to their wishes and needs [54, 57, 75]. Mobile health tools are supported ensuring flexible management through sensitization and optimizing the environment across multiple dimensions (individual, provider, and organizational levels) [54, 65]. Additionally, understanding common ground, exploring health and illness, valued customers, people-centredness, social and physical wellbeing and satisfaction, whole- PCC reported measures to improve health status and reduce morbidities and mortalities [51, 53, 58, 62]. Nonetheless, difficulties achieving mutual understanding between end users were influenced by several challenges such as lack of training and new skills of providers, lack of trust (genuine care, respect, dignity, autonomy), poor disclosure of problems (time-compressed visit) and lack of resources [60, 68, 79].

## Discussion

This review synthesizes evidence on people-centred PHC and primary care. Major themes identified from this review were community engagement, empowerment and empathy, leadership and mutual accountability within the organization, home and community-based and participatory care, holistic care for people with multimorbidity, partnership with information technology, coordination and communication, and flexible management for delivery of people-centred PHC services. Most studies in the HICs explained people-centred medical care models with little focus research in LMICs.

There are several ways that health systems could generate and deliver people-centred and integrated care for individuals, families, and communities. Firstly, promoting respectful conversations and activities between care providers and service users is fundamental for improving community empowerment and ensuring providers’ empathy. People engagement and empowerment enhanced people-centred PHC in many contexts. Empowering traditionally disengaged communities and individuals requires awareness of social determinants of health [80]. Conversation and engagement of people can support personalized, coordinated care towards narrowing inequalities [81]. The provider’s empathy also enabled supportive, involved care, community, social enterprise, and volunteerism [81]. Inter-professional teamwork and collaboration with and for older people and relatives are fundamental to empathy and empowerment [66]. Of the five strategies of the WHO framework on IPCHS, community engagement and empowerment have little attention in the literature. The current global health initiatives, including the Asthana Declaration, have envisioned empowerment, health literacy, and understanding the public’s role in PHC [82]; community engagement could potentially promote people-centred PHC service delivery. Thus, the focus of research, policy and practices of community engagement and empathy need to be prioritized in PHC and primary care in low-income settings.

Secondly, for PCC and coordinated care, there was an emphasis on organizational integrity and mutual accountability. Strengthening leadership and accountability in home-based care increased people-centred care in PHC services [83]. Co-creation and healthcare organizations and their leadership efficiently could meet the health needs of people according to standards of care to align tactics and improve organizational reliability while paying attention to quality care [84]. Organizational leadership and mutual accountability strategies could be beneficial in recruiting people with integrity and sensitivity, the ability to notice and respond through policies of diverse staff and aligning incentives and recognitions [11, 84].

Thirdly, some models of care, such as care for people with multiple chronic conditions or comorbidities, residential home-based care, and participatory care, were effective approaches for PCC in PHC and primary care contexts. Such care models can effectively reduce the burden of hospitalization and care costs by using PHC and primary care in prehospital settings [83, 85]. The residential home-based model of care facilitates holistic care through collaboration between family members and providers considering the family contexts and comprehensive education and care [86]. Such a model is useful for people with multiple chronic conditions that could support the activities of daily living and produce high healthcare expenses. Functional limitations can often complicate access to health care, interfere with self‐management, and necessitate reliance on caregivers [87]. Crucial for implementing people-centred care is knowing and confirming people as a whole and co-creating a tailored personal health plan [66]. These residential care models could enhance the identification of health priorities (i.e., specific health outcomes and healthcare preferences), and clinicians align their decision-making to achieve these health priorities [88].

Fourthly, partnership with the digital and information technology sector, and tools can potentially ensure coordinated care by monitoring health records, coordinating processes, tracking health services, and involving people representatives and individuals in developing digital services and work practices. The information technology-related stakeholders are vital for mutual information sharing and distributing initiatives, tasks, and responsibilities from providers to service users [89]. The human-centred service design approach can leverage the potential of technology and advance healthcare systems, and innovative solutions for healthcare change and wellbeing; addressing the complexity of healthcare systems toward integrated care [90].

Finally, enabling and flexibly managing the health system environment is fundamental for people-centredness in the provision of delivery of PHC services. System strengthening and management requires system inputs and processes towards desired outcomes. The structural factors of organizations and systems (e.g., creating a PCC culture across the continuum of care, co‐designing educational programs, health promotion and prevention programs with people) provide the foundation for PCC, providing a supportive and accommodating environment developing structures to support health information technology and measure and monitor people-centred care performance influence the processes and outcomes [91]. The processes component describes the importance of cultivating communication and respectful and compassionate care, engaging service users in managing care and integrating care. At the same time, outcome domains identified include access to care and client-reported outcomes [91]. At the system level, the enabling environment indicates the adaptation of responses, involvement in support, engagement with professionals, use of information and communication technologies, and organization of care [92].

This study has some limitations. We included studies written only in English. This study is a scoping review of qualitative evidence in the topic. We synthesized evidence rather than grading the quality of available evidence. Synthesized evidence from this study could provide research, policy, and program insights for improved people-centred PHC services. Evidence generated from this study is primarily based on studies from HICs and upper-middle-income countries (UMICs), which can have limited contextual implications in low-income countries as the health systems contexts of LMICs are different. Therefore, future research can be conducted on specific components of people-centred care in low-income country settings.

## Conclusions

Implementing several approaches of people-centred PHC and primary care, especially in HICs, has little priority in LMICs. Potential strategies for PCC could include engaging end users in the care process, community engagement and empowerment, mutual accountability, and institutional leadership. Some successful models of care, such as home-based residential care, are effective in care for people living with multimorbidity, and valuable in prehospital care that can reduce the care costs and burden to the health system. Flexible management options could create an enabling environment for health system strengthening in providing and delivering health services.

### Supplementary Information

**Additional file 1: Table S1.** Preferred Reporting Items for Systematic reviews and Meta-Analyses extension for Scoping Reviews (PRISMA-ScR) Checklist. **Table S2.** A summary of studies included in the review.

## Data Availability

All data generated or analyzed during this study are included in this published article [and its supplementary information files].
