# Supplementary material for: People-centred primary health care: a scoping review
Source: BMC Prim Care. 2023 Nov 9;24:236. doi: 10.1186/s12875-023-02194-3 (PMC10633931; doi:10.1186/s12875-023-02194-3)
Supplement: Supplementary file 1 — Additional file 1: Table S1. Preferred Reporting Items for Systematic reviews and Meta-Analyses extension for Scoping Reviews (PRISMA-ScR) Checklist. Table S2. A summary of studies included in the review. [file 12875_2023_2194_MOESM1_ESM.docx]

**Supplementary information**

**Table S1: Preferred Reporting Items for Systematic reviews and Meta-Analyses extension for Scoping Reviews (PRISMA-ScR) Checklist**

| **SECTION** | **ITEM** | **PRISMA-ScR CHECKLIST ITEM** | **Page #** |
| --- | --- | --- | --- |
| TITLE | | | |
| Title | 1 | Identify the report as a scoping review. | Page 1, |
| ABSTRACT | | | |
| Structured summary | 2 | Provide a structured summary that includes (as applicable): background, objectives, eligibility criteria, sources of evidence, charting methods, results, and conclusions that relate to the review questions and objectives. | Page 2 |
| INTRODUCTION | | | |
| Rationale | 3 | Describe the rationale for the review in the context of what is already known. Explain why the review questions/objectives lend themselves to a scoping review approach. | Page 4 |
| Objectives | 4 | Provide an explicit statement of the questions and objectives being addressed with reference to their key elements (e.g., population or participants, concepts, and context) or other relevant key elements used to conceptualize the review questions and/or objectives. | Page 4 |
| METHODS | | | |
| Protocol and registration | 5 | Indicate whether a review protocol exists; state if and where it can be accessed (e.g., a Web address); and if available, provide registration information, including the registration number. | NA |
| Eligibility criteria | 6 | Specify characteristics of the sources of evidence used as eligibility criteria (e.g., years considered, language, and publication status), and provide a rationale. | Page 5 |
| Information sources* | 7 | Describe all information sources in the search (e.g., databases with dates of coverage and contact with authors to identify additional sources), as well as the date the most recent search was executed. | Page 5 |
| Search | 8 | Present the full electronic search strategy for at least 1 database, including any limits used, such that it could be repeated. | Page 5 |
| Selection of sources of evidence† | 9 | State the process for selecting sources of evidence (i.e., screening and eligibility) included in the scoping review. | Page 6 |
| Data charting process‡ | 10 | Describe the methods of charting data from the included sources of evidence (e.g., calibrated forms or forms that have been tested by the team before their use, and whether data charting was done independently or in duplicate) and any processes for obtaining and confirming data from investigators. | Page 6 |
| Data items | 11 | List and define all variables for which data were sought and any assumptions and simplifications made. | Page 7, 11-16 |
| Critical appraisal of individual sources of evidence§ | 12 | If done, provide a rationale for conducting a critical appraisal of included sources of evidence; describe the methods used and how this information was used in any data synthesis (if appropriate). | NA |
| Synthesis of results | 13 | Describe the methods of handling and summarizing the data that were charted. | Page 6 |
| RESULTS | | | |
| Selection of sources of evidence | 14 | Give numbers of sources of evidence screened, assessed for eligibility, and included in the review, with reasons for exclusions at each stage, ideally using a flow diagram. | Page 7, flow chart |
| Characteristics of sources of evidence | 15 | For each source of evidence, present characteristics for which data were charted and provide the citations. | Page 7-19, references |
| Critical appraisal within sources of evidence | 16 | If done, present data on critical appraisal of included sources of evidence (see item 12). | NA |
| Results of individual sources of evidence | 17 | For each included source of evidence, present the relevant data that were charted that relate to the review questions and objectives. | Page 8-9 |
| Synthesis of results | 18 | Summarize and/or present the charting results as they relate to the review questions and objectives. | Page 9-19 |
| DISCUSSION | | | |
| Summary of evidence | 19 | Summarize the main results (including an overview of concepts, themes, and types of evidence available), link to the review questions and objectives, and consider the relevance to key groups. | Page 16 |
| Limitations | 20 | Discuss the limitations of the scoping review process. | Page 18-19 |
| Conclusions | 21 | Provide a general interpretation of the results with respect to the review questions and objectives, as well as potential implications and/or next steps. | Page 19 |
| FUNDING | | | |
| Funding | 22 | Describe sources of funding for the included sources of evidence, as well as sources of funding for the scoping review. Describe the role of the funders of the scoping review. | Page 19 |

Table S2: a summary of studies included in the review.

| **Authors** | **Aim** | **Country** | **Study type** |
| --- | --- | --- | --- |
| Scott et al | To define the joint principles of the patient-­‐centered medical home (PCMH) and describe the integration of pharmacists into a PCMH | US | Qualitative |
| Council et al | To describe how the adoption and use of a patient-centered care plan (PCCP) document enhanced care for complex patients and changed the relationships with health team members | US | Qualitative |
| Hudon et al | The objective was to provide a synthesis of the results of the research and discourse lines on main dimensions of patient-centered care in the context of chronic disease management in family medicine, building on Stewart et al.'s model. |  | Review |
| Leventhal et al | To demonstrate that concepts of patient-centeredness and technology-centeredness must work together within the context of the transformation to the patient-centered medical home (PCMH) | US | Qualitative |
| Reed et al | To understand the process of patient-centered care innovation undertaken by innovative health care organizations - from strategic planning to goal selection to implementation to maintenance. | US | Qualitative |
| Van Berckelaer et al | little is known about how patients view aspects of the PCMH or how they define patient-centeredness. | US | Qualitative |
| Lavoie et al | To explore dimensions identified as key in the patient-centred literature in the context of primary health care services delivered in a group setting | Canada | Qualitative |
| Rosstad et al | to develop pathways for patients diagnosed with heart failure, COPD and stroke. The aim of this paper is to investigate the process and the experiences of the participants in this developmental work. | Norway | Qualitative |
| Akinci and Patel | to breakdown and demonstrate the need for quality improvement in the U.S. delivery of healthcare by examining the PCMH. | US | Qualitative |
| Grace et al | To examine the facilitators and barriers of implementing a primary care team redesign intended to augment physician-medical assistant dyads by adding two new care team members | US | Qualitative |
| Mead et al | to investigate underserved, chronically ill patients' preferences for care and develops a patient-centered framework of priorities. | US | Qualitative |
| Flink et al | To explore patient handovers between primary and secondary care by assessing the levels of patient-centeredness of medical records used for communication between care settings and by assessing continuity of patient care | Sweden | Quantitative |
| Brown et al | to evaluate how these processes are enacted by 1 primary care model, Family Health Teams, in Ontario. | Canada | Qualitative |
| Doubova et al | study evaluated primary care attributes of patient-centered care associated with the public perception of good quality in Brazil, Colombia, Mexico and El Salvador. | Latin America. | Quantitative |
| Friedman et al | To describe experiences of care coordinators across the US from their own perspectives. | US | Qualitative |
| Hansen et al | To contribute to the debates about GPs and CPGs by reflecting on three different Australian studies conducted by the authors to better understand variations | Australia | Qualitative |
| Kogan et al | To describe PCC shifts, focus away from the traditional biomedical model in favour of embracing personal choice and autonomy for people receiving health services. | US | Review |
| Zhang et al | To describe electronic health records and their use may diminish "patient-centeredness" in exam rooms by distracting the healthcare provider from focusing on direct patient interaction. | US | Qualitative |
| Agha et al | To explore barriers to patient engagement efforts and their corresponding solutions. | US | Qualitative |
| Berntsen et al | To outline a 4-stage goal-oriented PC-IC process ideal | Norway | Qualitative |
| Poitras et al | to determine the elements of patient-centered interventions and interventions for persons with multimorbidity that are associated with positive health-related outcomes for patients. |  | Review |
| J. Reeve | Person-centred primary care is a priority for patients, healthcare practitioners and health policy. | UK | Qualitative |
| Karnimura et al | to examine factors that affect underserved primary care patients' perceptions of patient centeredness. | US | Quantitative |
| Kuipers et al | To describe the role of PCC in multi-morbidity poses a heavy burden on patients and is related to adverse outcomes. | Netherlands | Quantitative |
| Lionis et al | To focuses on the experiences from Greece in implementing primary health care reform and lessons learned from the conduct of evidence-based research. | Greece | Qualitative |
| Millenson et al | To examine four ACO innovators in patient-centered care that together represent urban, suburban and rural populations with a broad range of economic, racial, ethnic and geographic diversity. | US | Case Report |
| Petit-Steeghs et al | to analyze the perspectives of women on maternity care and to provide recommendations on how to achieve client-centred care. | The Netherlands | Qualitative |
| Santana et al | The shift to the patient-centred care (PCC) model as a healthcare delivery paradigm calls for systematic measurement and evaluation. |  | Review |
| Schimmer et al | to explore challenges that could impact the design of a person-centered eHealth service for T2D self-management support. | Sweden | Qualitative |
| Waweru et al | to identify relevant stakeholders' views on the current quality of primary health care services and their understanding of PCC | Uganda | Qualitative |
| Brickley et al | To synthesize literature investigating GP-delivered PCC and address 'what is currently known about GP-delivered PCC | Not specified | Review |
| Holt et al | To understand clinicians' perceived facilitators and barriers to use PCD using a consumer informatics technology integrated into the EHR. | Us | Qualitative |
| Kuipers et al | to identify views of patients with multi-morbidity on the relative importance of PCC aspects in a Dutch primary care setting | The Netherlands | Qualitative |
| Turner and Archer | to determine whether the Western constructs of empathy were relevant in our context | South Africa | Mixed Methods |
| Waweru et al | To describe the patient communication with their doctors and understand the cause of their problems as well as the management plan | Uganda | Mixed Methods |
| Ahmed et al | to identify the relationships between the context in which integrated care programmes (ICPs) for community-dwelling frail older people |  | Review |
| Brault et al | To explore facilitators and barriers to patient-centered communication in the context of developing an electronic appointment planning tool to promote SRH communication in clinic settings | US | Qualitative |
| Brickley et al | to evaluate and advance a theoretical model of PCC developed in consultation with practising GPs and patient advocates. | Australia | Qualitative |
| Ebrahimi et al | to explore the content and essential components of implemented person-centered care in the out-of-hospital context for older people |  | Review |
| Epstein et al | To explore patients' and GPs' perceptions of an alternative payment system, a Patient-Chosen Gap Payment, | Australia | Qualitative |
| Kuipers et al | To healthcare professionals' perceived barriers to primary PCC delivery to patients with multimorbidity were investigated | The Netherlands | Qualitative |
| Kumar et al | To encompasses an overview of mental health care competencies, best practices and capacity building needed to fast track patient responsive |  | Narrative Synthesis |
| Raj et al | To examine what patient-centeredness means for older adults and family caregivers, and (2) assess circumstances underlying their preference for geriatric care. | US | Qualitative |
| Westlake et al | To explain the engagement and understanding from the patient, a PCC approach will fail to initiate. | England | Qualitative |
| Armenta-Arellano et al | to materialize the right to health in accordance with its laws. | Mexico | Qualitative |
| Aworinde et al | To identify published evidence on person-centered outcome measures (PCOMs) used in dementia care and to explore how PCOMs facilitate shared decision-making and improve outcomes of care. | Not specified | Mixed Methods -Review |
| Kari et al | to develop an interprofessional people-centred care model (PCCM), including the contribution of a clinically trained pharmacist for home-living multimorbid older people in primary care. | Finland | Qualitative |
| Lalani and Marshall | To explain consider the extent to which co-location is an enabler for service integration by examining multi-professional community care teams. | England | Qualitative |
| Lateef and Mhlongo | to explore the perception of nurses on PCC | Nigeria | Qualitative |
| Manalili et al | to contribute to developing practical guidance for implementing person-centred quality indicators (PC-QIs) for primary care | Canada | Qualitative |
| Schuttner et al | to clarify facilitators and barriers perceived by primary care physicians in the Veterans Health Administration to delivering patient-centered care for high-risk or complex patients with multimorbidity. | US | Qualitative |
| Tuepker et al | To explore the understanding how these services are being used or experienced by this population. | US | Qualitative |
